# From tissue-specific to tissue-agnostic: HER2 overexpression and the rise of antibody-drug conjugates

**DOI:** 10.3389/fonc.2025.1565872

**Published:** 2025-03-03

**Authors:** Heidi C. Ko, Kyle C. Strickland, Dana Jaggessarsingh, Alicia Dillard, Michelle Green, Erin Newburn, Tiffany Sellaro, Jonathan L. Klein, Brian Caveney, Marcia Eisenberg, Eric A. Severson, Shakti Ramkissoon, Rebecca A. Previs, Anis Toumeh

**Affiliations:** ^1^ Labcorp, Durham, NC, United States; ^2^ Department of Pathology, Duke University Medical Center, Duke Cancer Institute, Durham, NC, United States; ^3^ Labcorp, Shelton, CT, United States; ^4^ Wake Forest Comprehensive Cancer Center and Department of Pathology, Wake Forest School of Medicine, Winston-Salem, NC, United States; ^5^ Division of Gynecologic Oncology, Department of Obstetrics & Gynecology, Duke University Medical Center, Duke Cancer Institute, Durham, NC, United States; ^6^ Hospital Corporation of America (HCA) Midwest Oncology Associates, Overland Park, KS, United States

**Keywords:** HER2 overexpression, *ERBB2* amplification, pan-tumor testing, antibody-drug conjugate, next-generation sequencing, tissue stewardship, precision oncology, tumor-agnostic biomarker

## Abstract

The growing importance of HER2 expression as a biomarker across multiple cancers is largely driven by advances in HER2-directed antibody-drug conjugates. The recent approval of trastuzumab deruxtecan (T-DXd) as a tumor-agnostic therapy has revolutionized treatment strategies for HER2-overexpressed tumors beyond breast, gastric, and colorectal cancers (CRC). This mini-review explores the evolving role of assessing HER2 overexpression in pan-solid tumors, following the recent approval of T-DXd as a tumor-agnostic therapy. It examines how HER2 scoring criteria for pan-tumor indications rely on immunohistochemistry (IHC) assessment, which may be prone to subjective interpretation and interobserver variability, and how these criteria differ from those used in breast, gastric, and CRC tumors. We also address the potential for NGS approaches to identify *ERBB2* copy number gain (CNG) and the utility of artificial intelligence (AI) algorithms to enhance the consistency and accuracy of HER2 score interpretation for T-DXd treatment eligibility in solid tumors.

## 1 Introduction

Human epidermal growth factor receptor 2 (HER2) is a member of the epidermal growth factor receptor (*EGFR*) family of transmembrane glycoproteins with tyrosine kinase activity which are essential in regulating epithelial cell proliferation, differentiation, and survival (1). Abnormal activation of HER2 activity can occur through amplification or mutation of the *ERBB2* gene, leading to dysregulated cell growth and tumorigenesis. *ERBB2* amplification is a more commonly observed alteration event which is associated with overexpression of HER2 protein, triggering oncogenesis and uncontrolled cell growth through PI3K/AKT/mTOR and MAPK signaling pathways in many cancers (2).

HER2 overexpression is clinically assessed using an immunohistochemistry (IHC) staining assay, with scores ranging from very low or absent (0) to very high (3+). HER2 IHC score of 0 represents a staining pattern where there are fewer than 10 percent of tumor cells with no staining or incomplete membrane staining that is faint or barely perceptible. A score of 1+ is defined as faint or barely perceptible incomplete membrane staining in more than 10 percent of tumor cells. Tumors with weak-moderate complete membrane staining in more than 10 percent of tumor cells are scored as 2+. Lastly, tumors with complete, intense membrane staining involving more than 10 percent of tumor cells are scored as 3+. In cases of equivocal IHC score of 2+ in breast, gastric, and colorectal cancers, reflex *in-situ* hybridization (ISH) is performed to confirm *ERBB2* amplification (3–5).

HER2 overexpression plays a critical role in oncogenesis of breast tumors, arising in almost half of *in-situ* carcinomas and 20% of invasive breast cancers (1). Beyond breast cancer, *ERBB2* amplification and HER2 overexpression are observed across other various tumor types such as gastric, bladder, colorectal, bile duct, and non-small cell lung cancers (2, 6). Overexpression of HER2 suggests an aggressive tumor phenotype and a historically unfavorable prognosis in many cancers (1, 2). However, the development of HER2-targeted therapies has significantly improved the prognosis and survival of patients with HER2-overexpressed cancers, particularly in breast cancer (2). Historically, mechanisms of HER2-targeted therapies included HER2 monoclonal antibodies (mAb) and tyrosine kinase inhibitors (TKIs). In more recent years, anti-HER2 antibody-drug conjugate (ADCs) have emerged as a new standard-of-care treatment for patients with HER2-overexpressed or mutated cancers (2).

## 2 Structure and function of antibody-drug conjugates (ADCs) targeting HER2

ADCs are usually comprised of a tumor antigen-selective mAb covalently attached to a highly potent cytotoxic chemotherapy via a chemical linker (7). The construct of an ADC enables precise delivery of the cytotoxic chemotherapy to the tumor, leading to potent killing of the cancer cells while minimizing the off-target systemic drug toxicities to healthy tissues (7–9).

The identification of HER2 as a therapeutic target in the 1980’s paved the way for development of HER2-targeted ADCs as treatments for HER2-altered cancers (10). All the currently available HER2-targeted ADCs use trastuzumab as the mAb, which was the first fully humanized mouse antibody to be approved by the FDA in 1998 (11). Trastzumab emtansine (T-DM1) is a HER2-directed ADC that combines trastuzumab with a cytotoxic microtubule inhibitor, called emtansine (DM1) via an uncleavable linker (12). In the phase III EMILIA trial, treatment with T-DM1 led to significant improvements in objective response rates (ORR), progression-free survival (PFS), and overall survival (OS) in patients with HER2-overexpressed metastatic breast cancer who previously failed trastuzumab and chemotherapy as compared with capecitabine plus lapatinib (13).

Trastuzumab deruxtecan (T-DXd) is a newer generation HER2-directed ADC which has shown superior therapeutic efficacy to T-DM1 in clinical trials (14). T-DXd is an ADC that consists of trastuzumab and a topoisomerase I inhibitor, DX-8951. T-DXd has unique properties that increase its potency over T-DM1. First, T-DXd contains a higher cytotoxic drug-to-antibody ratio of 8:1 as compared with the 3.5:1 in T-DM1 (12, 15). Second, T-DXd has higher membrane permeability that allows for improved stability in the plasma until it is internalized into the target cells. Third, the linker that attaches the chemotherapy payload to trastuzumab is cleavable, enabling more efficient processing and release of the cytotoxic drug into the cell (12). Finally, once T-DXd is internalized and processed by the target cells, it has a unique ability to enter the surrounding cells and induce a bystander killing effect to the off-target cancer cells, maximizing its therapeutic efficacy even in tumors with heterogeneous expressions of HER2 (12, 14). These characteristics of T-DXd enable potent delivery of the chemotherapy to the cancer cells, resulting in cell cycle arrest and apoptosis (12). The unique properties of T-DXd have broadened its therapeutic efficacy in tumors with lower HER2 expression, classified as HER2-low (IHC 1+ or 2+/ISH-negative) and ultralow (IHC 0 with membrane staining) breast cancer (16, 17). These tumors were previously considered HER2-negative and did not have therapeutic responses to anti-HER2 mAbs and T-DM1 (18, 19).

## 3 Spotlight on T-DXd as treatment for HER2-altered cancers

### 3.1 Breast cancer

Early clinical success of T-DXd in solid tumors was established through breast cancer-specific studies, notably DESTINY-Breast01 and 02 trials. Treatment with T-DXd resulted in an ORR of 60.9% (20) and led to an improvement in PFS by 64% (21) in patients with unresectable or metastatic HER2-overexpressed, defined as IHC 3+ or 2+/ISH-positive, breast cancer after progression on prior treatments including anti-HER2 therapies. In 2022, T-DXd further demonstrated therapeutic efficacy in patients with intermediate to low levels of HER2 expression, defined as HER2-low tumors with IHC 1+ or 2+/ISH-negative scores (16). These practice-changing data were reflected in the DESTINY-Breast04 trial, where the risk of disease progression was 50% lower and the risk of death was 36% lower in patients treated with T-DXd compared to chemotherapy, regardless of hormonal receptor status (16). Ultimately, these results led to the U.S Food and Drug Administration (FDA) approval of T-DXd for the treatment of advanced HER2-low breast cancer. More recently, the DESTINY-Breast06 phase III clinical trial demonstrated clinically meaningful activity of T-DXd in chemotherapy-naive patients with even lower levels of HER2 expression, defined as HER2 ultralow (IHC 0 with membrane staining) breast cancer with an improvement in PFS of 22% as compared to chemotherapy (22). Following the positive results benefiting a broader patient population with lower levels of HER2 expression, the U.S FDA has approved T-DXd for the treatment of chemotherapy-naïve patients with unresectable or metastatic HER2-low (IHC 1+ or 2+/ISH-) or HER2-ultralow (IHC 0 with membrane staining) breast cancer after at least one line of endocrine therapy (23–25).

### 3.2 Gastric cancer

The phase II DESTINY-Gastric01 was the first study that showed the therapeutic efficacy of T-DXd in patients with HER2-overexpressed advanced gastric cancer who have received at least two prior lines of therapy including trastuzumab. HER2 overexpression was defined as IHC 3+ or IHC 2+/ISH+ for *ERBB2* amplification (26). Treatment with T-DXd led to an ORR of 51% and a median OS of 12.5 months as compared to an ORR of 14% and a median OS of 8.4 months with chemotherapy (26). Based on these results, the U.S FDA approved T-DXd for the treatment of unresectable and/or metastatic HER2-overexpressed gastric or gastroesophageal junction (GEJ) adenocarcinoma after progression on a prior trastuzumab-based regimen. Additional studies are ongoing to further evaluate the efficacy and safety of T-DXd, either alone or in combination with other therapies, for the treatment of HER2-overexpressed advanced gastric or GEJ adenocarcinoma. An ongoing phase III DESTINY-Gastric04 trial is evaluating T-DXd versus traditional chemotherapy-based regimen in patient with HER2-overexpressed gastric or GEJ adenocarcinoma after progression on trastuzumab-based therapy (27).

### 3.3 Colorectal cancer (CRC)

*ERBB2* amplification or HER2 overexpression is detected in about 2% of all CRC and represents a therapeutic target for treatment eligibility with HER2-targeted therapies (28). Initial HER2-targeted therapies for CRC centered on dual inhibition with HER2-directed mAbs and tyrosine kinase inhibitors in patients with HER2-overexpressed tumors, defined as IHC 3+ or IHC 2+ with *ERBB2* amplification on ISH (29). More recently, HER2-directed ADCs have emerged as a therapeutic option for those patients with HER2-overexpressed CRC. Although results from the HERACLES B study exploring the efficacy of T-DM1 in HER2-overexpressed CRC were not significant (30), T-DXd demonstrated clinically meaningful benefits in patients with HER2-overexpressed (IHC 3+) CRC after two prior lines of therapy including trastuzumab and pertuzumab (31). Treatment with T-DXd resulted in an ORR of 45.3% in patients with previously treated HER2-overexpressed CRC (28, 31). Given its clinical efficacy and tolerable safety profile, T-DXd has been approved by the U.S FDA for the treatment of HER2-overexpressed (IHC 3+) CRC.

### 3.4 Non-small cell lung cancer (NSCLC)

In NSCLC, T-DXd is approved for use in patients with *ERBB2*-mutant as well as HER2-overexpressed tumors (IHC 3+) unresectable or metastatic disease. A multi-cohort DESTINY-Lung01 trial showed that treatment with T-DXd resulted in a promising antitumor activity with a confirmed ORR of 55% in patients with previously treated *ERBB2*-mutant NSCLC (32). A separate cohort of DESTINY-Lung01 demonstrated results of ORR 34% in patients with HER2-overexpressed NSCLC (33).

### 3.5 All other solid tumors

In an effort to expand T-DXd use across all solid tumors, the recent phase II DESTINY-PanTumor02 trial evaluated its efficacy in HER2-overexpressed (IHC 3+/2+ with ISH positivity), unresectable locally advanced or metastatic solid tumors upon disease progression on first line therapy (34). Eligible patients had solid tumors ranging from biliary tract, bladder, cervical, endometrial, ovarian, pancreatic to other solid cancers that were not breast, colorectal, gastric, or NSCLC. At the primary analysis, the study investigators found that treatment with T-DXd achieved clinically significant results with ORR of 37.1% in all patients and 61.3% in those whose tumors had HER2 IHC 3+ expression. The median duration of response was 11.3 months in all patients and 22.1 months in those with HER2 IHC 3+ tumors (34). Given these encouraging results, the U.S. FDA recently granted an accelerated approval to T-DXd for patients with unresectable or metastatic relapsed HER2-overexpressed (IHC 3+) solid tumors (35). This approval represents the first tumor-agnostic approval for an antibody-drug conjugate and introduces IHC testing as a tumor-agnostic biomarker assessment for solid tumors.

## 4 Discussion

Although HER2 expression has evolved into a tumor-agnostic biomarker with therapeutic implications across all solid tumors, there are practical considerations around this newly defined tumor-agnostic biomarker.

### 4.1 Practical considerations around HER2 testing as a tissue agnostic biomarker

**Figure 1:**
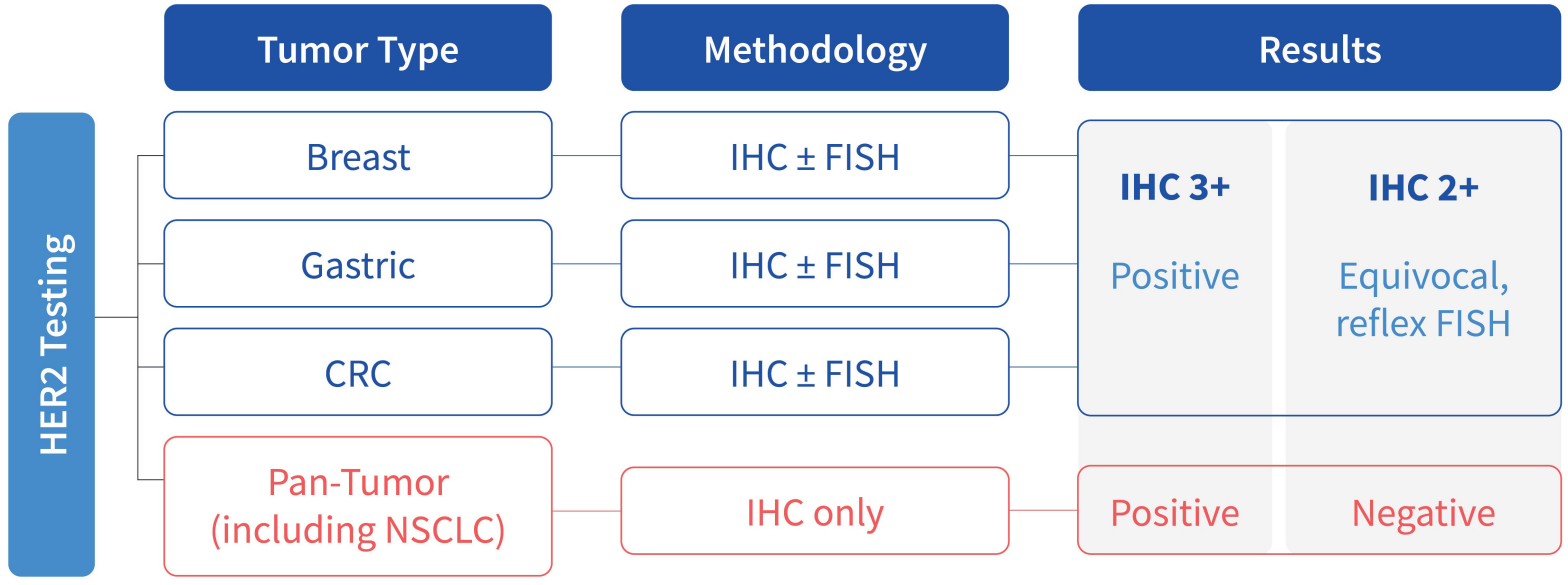
Scoring criteria for HER2 overexpression across various tumor types.

Several pathological and laboratory considerations have emerged since the DESTINY-PanTumor02 trial and subsequent FDA tumor-agnostic approval for T-DXd. First and foremost, HER2 overexpression criteria (IHC 3+), set in DESTINY-PanTumor02, differs significantly from traditional tumor types such as breast, gastric/GEJ, and colorectal cancers (**Figure 1**) (34, 36). Traditional HER2 testing protocols, developed for breast and gastric cancers and adopted for colorectal cancer, allow for HER2 assessment via IHC followed by reflex to *in situ* hybridization (ISH) for equivocal (IHC 2+) cases (36). In contrast, recent FDA approval for T-DXd as a tumor-agnostic indication is based on a HER2 IHC 3+ score and does not require ISH confirmation of equivocal cases by IHC (**Figure 1**). Therefore, while HER2 tumor-agnostic indication expands treatment options for solid tumors, it is critical for laboratories to maintain tumor-specific testing protocols and resulting to ensure appropriate therapeutic decisions.

**Figure 2:**
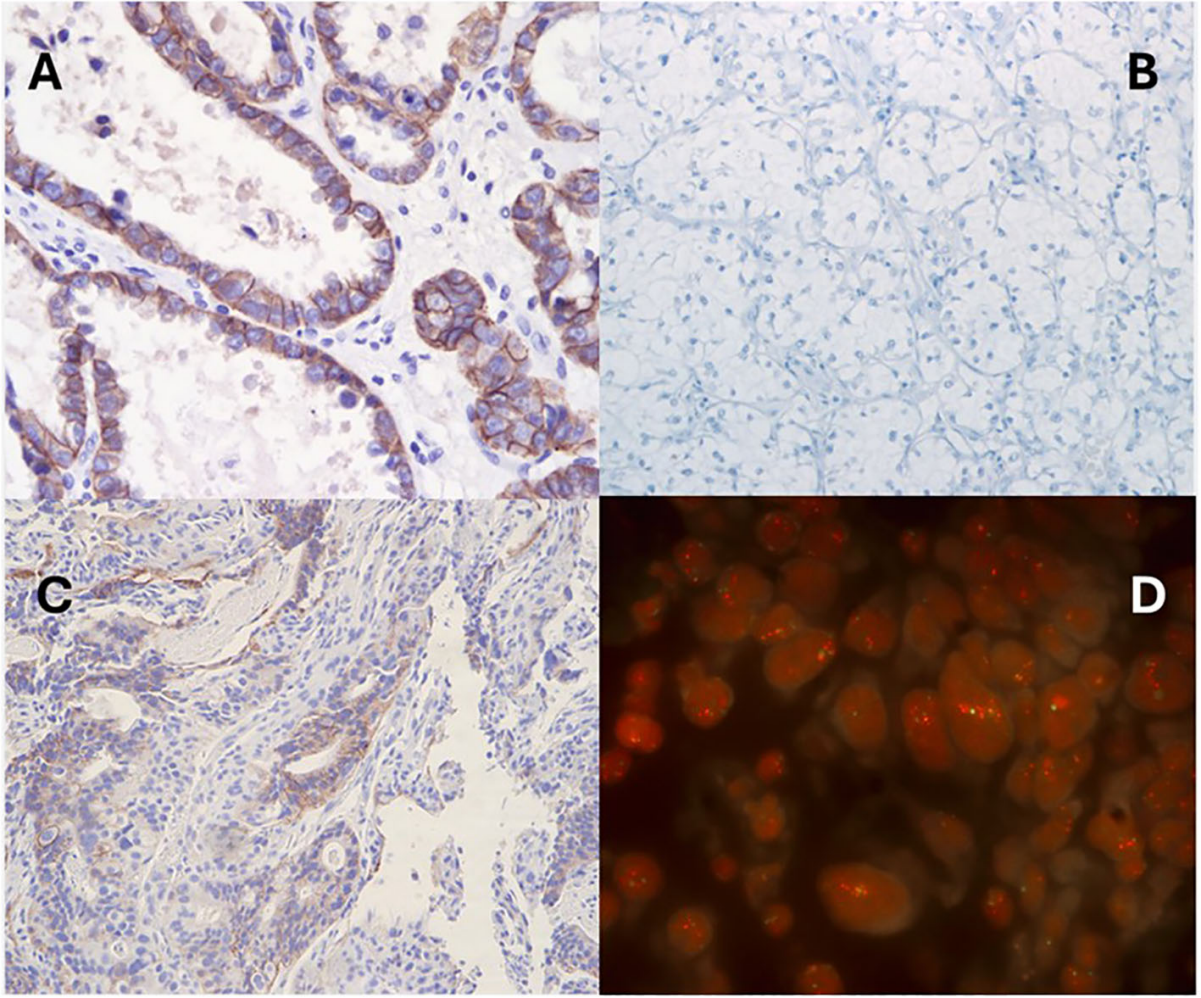
Representative results of HER2 immunohistochemistry and fluorescence *in situ* hybridization in solid tumors. **(A)** ovarian clear cell carcinoma with IHC 3+, **(B)** clear cell renal cell carcinoma with IHC 0, **(C)** gastroesophageal adenocarcinoma with IHC 2+ with FISH positivity **(D)**.

Second, most tumor-agnostic approvals are dependent on a binary assessment of either the presence or absence of a biomarker, such as TRK inhibitor eligibility for those patients with *NTRK* fusion-positive solid tumors (37). However, treatment eligibility for T-DXd in solid tumors relies on a more subjective approach of assessing tumor HER2 expression via IHC assay, subject to interobserver variability among pathologists. In addition, HER2 staining intensity may be influenced by various laboratory factors, including sample storage conditions, tissue section thickness, buffer solutions, incubation durations, variations among commercially available antibodies, control tissue selection, and quality control practices. **Figure 2** showcases real-world stained images of HER2 IHC and ISH in solid tumors. While the inter-observer agreement is generally better at differentiating IHC 0 and 1+ from 3+ cases, high interobserver variability can occur in discerning 2+ from 3+ scores, which can ultimately influence the eligibility for tumor-agnostic therapy (38, 39).

Third, specific criteria for HER2 expression and patient selection for T-DXd eligibility should be carefully reviewed. DESTINY-PanTumor02 trial allowed enrollment of patients with HER2 IHC 2+/ISH+ and IHC 3+ tumors. However, patients with biliary tract cancers with IHC 2+/ISH+ did not have meaningful treatment responses to T-DXd while patients with HER2 IHC 2+/ISH+ gynecologic cancers had high response rates (34). Based on the consistent results across tumors with IHC 3+ score, the FDA approval recognizes the HER2 overexpression as IHC 3+ for treatment eligibility with T-DXd as a tumor-agnostic therapy. However, additional studies might be warranted in certain tumor types such as endometrial cancer to determine if expanded populations of patients with lower HER2 expression might benefit from T-DXd. In addition, while T-DXd is approved for pan-solid tumor indications, certain tumor types such as sarcomas and renal cell carcinoma were not represented in the DESTINY-PanTumor02 trial (34). As a result, careful patient and tumor selection is essential to minimize the risk of harm and toxicity in those unlikely to benefit from T-DXd.

### 4.2 Future directions

Efforts are ongoing to develop a more precise HER2 diagnostic test to accurately identify patients who could benefit from T-DXd (40, 41). Next-generation sequencing (NGS) has become integral in management of advanced cancers as options for targeted therapies have expanded in oncology. The application of NGS can also facilitate effective tissue stewardship to ensure sufficient material is available to assess all clinically relevant biomarkers in a single test.

Regarding *ERBB2*/HER2 alterations, NGS has been utilized primarily to identify activating *ERBB2* (HER2) mutations, which have therapeutic implications in NSCLC. However, using tissue- or plasma-based NGS testing to detect *ERBB2* copy number gain (CNG) as a predictor of response to anti-HER2 therapy is not yet standard practice. The correlation between *ERBB2* CNG identified through NGS and HER2 overexpression determined by conventional IHC testing remains less well established. Although *ERBB2* CNG is not currently included in the FDA tumor-agnostic approval for HER2 testing, studies have demonstrated high concordance between NGS-detected *ERBB2* CNG and HER2 overexpression by traditional IHC/ISH methods, suggesting that NGS could serve as a reliable alternative or surrogate in certain cases (42–44). A recent multi-center phase II basket study has shown that treatment with T-DXd resulted in high ORR (56.5%) with durable responses in patients with advanced *ERBB2*-amplified solid tumors, detected by cell-free DNA (cfDNA) NGS testing (45). This study suggests that detection of *ERBB2* CNG through plasma-based ctDNA NGS assay could potentially serve as an alternative tool for determining T-DXd treatment eligibility in the future, especially when tissue availability is limited. However, NGS does not detect protein overexpression directly, and its results should be interpreted in conjunction with clinical findings and other diagnostic tests.

There have also been developments of artificial intelligence (AI) algorithms to improve the interpretation of HER2 IHC scores to be more consistent and accurate among pathologists (41, 46). Krishnamurthy et al. demonstrated that a fully automated AI tool led to increased interobserver agreement (75% manual to 83.7% AI-assisted review) and scoring accuracy (85.3% manual to 88% AI-assisted review) of HER2 IHC by pathologists (46). Among the cases of HER2 IHC 0 and 1+ cases, AI-assisted review led to even higher interobserver agreement (69.8% manual vs. 87.4% AI-assisted review) and accuracy (81.9% manual vs. 88.8% assisted review) (46). Although T-DXd is currently indicated for pan-solid tumors with HER2 overexpression (IHC 3+), data from breast cancer studies suggest it may also be effective in tumors with lower HER2 expression levels (IHC 1+ or 0), where AI tools can enhance the consistency and accuracy of HER2 interpretation.

## 5 Conclusion

The DESTINY-PanTumor02 trial and the subsequent approval of T-DXd have revolutionized the management of HER2-overexpressed solid tumors. It also represents the first tumor-agnostic therapeutic approach that leverages a protein biomarker overexpression and has expanded therapeutic opportunities across multiple cancer types. This evolving landscape of T-DXd eligibility reinforces the need for comprehensive biomarker assessment in solid tumors while balancing the increasing demand for molecular testing and the constraints of limited tissue samples in precision oncology.
